# The Domestication and Cultivation of *Pholiota adiposa* and Its High-Temperature Adaptability: Enhancing the Utilization of Agricultural Residues and Grain Nutrition in Northeast China

**DOI:** 10.3390/foods14101779

**Published:** 2025-05-16

**Authors:** Hu Lou, Baozhen Fan, Chao Guo, Yurong Liang, Weizhi Wang, Enze Yu, Jie Zhang, Guocai Zhang

**Affiliations:** 1 College of Life Science, Northeast Forestry University, 26 Hexing Road, Harbin 150040, China; longglehu@126.com (H.L.); fantexi0820@163.com (B.F.); gc1134393183@nefu.edu.cn (C.G.); yu2137163034@163.com (E.Y.); 2 College of Forestry, Northeast Forestry University, 26 Hexing Road, Harbin 150040, China; lyr9413@163.com; 3 Laboratory of Stress Response Biology, Graduate School of Science, Kyoto University, Kyoto 606-8317, Japan; weizhi66666@gmail.com; 4 Key Laboratory of Saline-Alkali Vegetation Ecology Restoration, Ministry of Education, Northeast Forestry University, 26 Hexing Road, Harbin 150040, China

**Keywords:** edible mushroom, *Pholiota adiposa*, mycelium material, agricultural and forestry waste, solid-state fermentation

## Abstract

*Pholiota adiposa* is a macrofungi that is rich in nutrients and has a delicious taste. Eating more can improve human immunity and inhibit cancer. However, the *P. adiposa* yield is low and cannot meet market demand. Therefore, strain improvement was carried out by exploring the mechanism of stress adaptation in *P. adiposa*. In addition, fermentation of the four common grains by *P. adiposa* mycelia increased their nutrient content and improved their antioxidant capacity. The results revealed that the growth of the mycelium was greatest when sucrose was used as the carbon source at 25 °C. At 35 °C, the MDA content and cellulase enzyme activity of the mycelia decreased by 27.6% and 40.8%, respectively, from 2 to 4 days, and the SOD, CAT, and GR enzyme activities increased by 31.6%, 49.2%, and 1.2%, respectively. The fermentation results revealed that the soluble protein content, reducing sugar content, and DPPH free radical scavenging ability of the fermented grains were significantly greater than those of the unfermented grains. This study can be used to cultivate macrofungi with environmental adaptability and provides a basis for the utilization of biological waste and increased food nutrition.

## 1. Introduction

*Pholiota adiposa* is a well-known macrofungi of Basidiomycota, Strophariaceae, and *Pholiota* [1]. It is rich in protein, polysaccharides, amino acids, vitamins, and trace elements and has a low fat content [2]. The surface of *P. adiposa* produces a large amount of mucus, which is a mixture of nucleic acid and mucopolysaccharide and has the effects of restoring human energy and improving brain rest [3]. *P. adiposa* is widely distributed in China, Japan, Europe, North America, and other places and is more common in Northeast China. It is mostly grown on dead trees, fallen trees, and stumps of broad-leaved forests such as willows and birches and mainly grows in forests below 1500 m above sea level [4,5].

The active substances in *P. adiposa* have antibacterial effects, lowering blood pressure, preventing gastric ulcers, exerting antitumor effects, and treating diabetes [6,7]. Studies have shown that the methanol extract of *P. adiposa* has strong antibacterial activity and that its polysaccharides also have antitumor and antidiabetic effects [8]. In addition, fermented broth was used to develop beverages, and the fermented products were added to produce functional rice wine and cans with the ability to treat hypertension. The use of *P. adiposa* in food production is also very extensive [9].

Temperature is one of the key factors affecting the growth and development of *P. adiposa*. Many edible and medicinal fungi have low temperature requirements [10]. In addition to differences in the optimum temperature for mycelial growth, many macrofungi need a certain temperature difference during growth and development to stimulate the formation of fruiting body primordia [11]. However, in the process of cultivation, the accumulation of bacterial bags produces a high-temperature environment, and the growth of fruiting bodies is highly susceptible to high temperatures [12]. A temperature that is too high will change the activity of biochemical enzymes, reduce the respiration rate, and affect yield. High temperature can also destroy the cell structure of *P. adiposa*, inhibit the growth of the mycelium, affect cell metabolism, and reduce quality and yield [12,13]. Therefore, exploring the effects of high-temperature stress on *P. adiposa* can provide solutions for addressing high-temperature stress during cultivation.

Solid-state fermentation (SSF) is a process in which microorganisms ferment on solid substrates with or without free water. It is widely used in the production of rice wine, wine, vinegar, and soy sauce [14]. The production of bread, cheese, and alcoholic beverages using fungal mycelium-fermented cereals can increase taste and nutrient content [15]. However, solid-state fermentation technology for mycelia is not mature. Therefore, the use of solid-state fermentation technology to ferment corn, soybean, wheat, and rice and determine nutrient contents can significantly improve the nutrient content and antioxidant capacity of cereals.

In this study, we domesticated and cultivated *P. adiposa* from the wild. By exploring the effects of temperature and moisture on the growth of *P. adiposa*, we found that the most suitable cultivation material was sawdust with corn straw. The optimum growth conditions for mycelia were 25 °C, the pH was 6, and sucrose was used as the carbon source. In addition, we improved the strain by exploring the mechanism of adaptation to stress and explored the application of *P. adiposa* mycelia in grain fermentation. Our study confirmed that the lipid scale umbrella we studied is an excellent strain that can resist high-temperature stress and significantly increases the soluble protein content, reducing sugar content and DPPH (1,1-diphenyl-2-picrylhydrazyl radical) free radical scavenging activity of fermented grains. This study can be used to develop environmentally adaptable edible and medicinal macrofungi, providing a basis for the utilization of biological waste and increasing food nutrition.

## 2. Materials and Methods

### 2.1. Sample Collection, Separation, and Pure Culture

**Figure 1:**
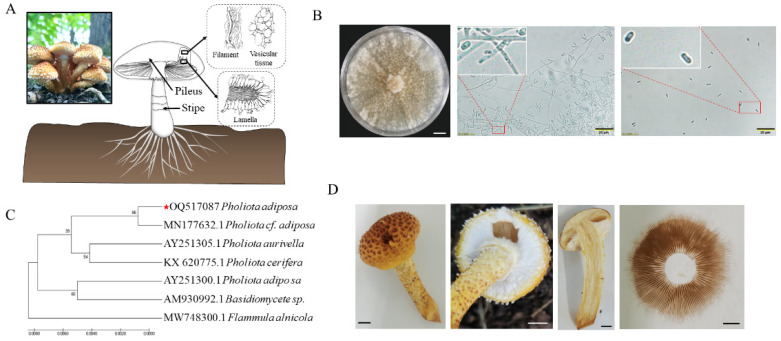
The pure culture and morphological characteristics of *P. adiposa* mycelia. (**A**) The structural diagram of the multiscale umbrella. (**B**) The separation and purification of *P. adiposa* mycelia. (**C**) Phylogenetic trees constructed for *P. adipos*. (**D**) Fruiting bodies and spore prints of *P. adiposa*. (**B**) Scale bar = 20 μm, (**D**) Scale bar = 1 cm.

The sampling site was a mixed forest ecosystem predominantly composed of *Quercus mongolica* and *Betula platyphylla* at the forest farm of Northeast Forestry University, which was established in 2003 and encompasses an area of 43.95 hectares (126°37′49.67″ E, 45°43′24.75″ N, Harbin, China) (left side of Figure 1A). The fungal material (*P. adiposa*) was collected from the decaying wood and forest floor within this mixed forest environment. After tissue isolation, the fruiting bodies of *P. adiposa* were purified many times to obtain the strain (GenBank accession number: OQ517087). The purification process for the specific strain was as follows: the soil and other impurities on the surface of *P. adiposa* were removed with a soft brush, the sample was rinsed under water for 2 min, and the *P. adiposa* sample was subsequently dried with sterile filter paper. The samples were soaked in alcohol (LIRCON, Dezhou, China) for 30 s and rinsed with sterile water 5 times, after which the surface moisture was removed by drying with sterilized qualitative filter paper. Approximately 0.2 cm of tissue from the cross section of the junction of the pileus and the stipe of the fruiting body were cut and placed in PDA media (200 g of potato boiled for 15 min, 4 layers of gauze filtration, 20 g of glucose, 3 g of KH_2_PO_4_, 1.5 g of MgSO_4_·7H_2_O, 18 g of agar (SCR Co., Ltd., Shanghai, China), and 1000 mL of distilled water). The PDA plate was cultured in an incubator (Zanwei Co., Ltd., Shanghai, China) at 25 °C in the dark. The mycelia were repeatedly purified after they were grown on PDA plates, after which the *P. adiposa* strain was obtained.

### 2.2. DNA Extraction, Sequencing, and Phylogenetic Analysis

The CTAB method was used to extract DNA from the mycelia. Specifically, *P. adiposa* was inoculated into PDA liquid media and cultured for 7 days to obtain mycelia (at 25 °C and 130 rpm·min^−1^). The powder was ground in liquid nitrogen, and 500 μL of 1% CTAB (SCR Co., Ltd., Shanghai, China) preheated at 65 °C was added. The mixture was subsequently heated for 65 min. Then, 50 μL of the mixture (chloroform/isoamyl alcohol = 24:1) (SCR Co., Ltd., Shanghai, China) was added, and the mixture was mixed well and left for 10 min (12,000 rpm·min^−1^). The supernatant was added to 500 μL of precooled isopropanol and allowed to stand on ice for 5 min. The samples were subsequently centrifuged for 10 min, 500 μL of 75% precooled ethanol (SCR Co., Ltd., Shanghai, China) was added, and the samples were subsequently centrifuged for 10 min (12,000 rpm·min^−1^). The DNA precipitate was dried at room temperature, and 50 μL of deionized water was added for use.

The PCR system (20 μL) was as follows: 10 μL of enzyme mixture, 0.8 μL of primer ITS 1/ITS 4, 1 μL of DNA template (TAKARA, Dalian, China), and 7.4 μL of ddH_2_O. The PCR procedure was as follows: predenaturation at 94 °C for 5 min; denaturation at 94 °C for 30 s; annealing at 57 °C for 30 s; extension at 72 °C for 1.5 min; and 72 °C for 10 min for a total of 35 cycles. The purity of the PCR products was detected by 1% agarose gel (Biowest, Rue de la Caille, France) electrophoresis. After electrophoresis, the products were observed under a UV imager (Saizhi Co., Ltd., Beijing, China). The PCR products were sent to Jilin Province Kumei Biotechnology Co., Ltd. (Guiyang, China), for sequencing, and the sequencing results were compared with those in the NCBI database via the Blast program (GenBank accession number: OQ517087). The phylogenetic tree was constructed via MEGA 7.0 software.

### 2.3. Study of the Different Growth Conditions of P. adiposa Mycelia

The effects of different carbon sources in the media on the growth of the mycelia were as follows: 2 g of peptone, 1.5 g of MgSO_4_·7H_2_O, 3 g of K_2_HPO_4_, and 18 g of agar (Saizhi Co., Ltd., Beijing, China). Different carbon sources were subsequently added according to the proportion of medium: 19 g of sucrose, 18 g of soluble starch, 19 g of maltose, 19 g of lactose, and 20 g of fructose (Saizhi Co., Ltd., Beijing, China) were added. The solution was subsequently diluted to 1 L with distilled water. A 6 mm diameter block was inoculated on different carbon-source solid plate media and cultured in a 25 °C static incubator (Zanwei Co., Ltd., Shanghai, China) under dark conditions until one of the treatment groups was covered with a plate. Each treatment group had 3 replicates. The colony diameter was measured via the cross-crossing method: a cross line was drawn along the center of the inoculation point on the back of the culture dish, and the colony diameter was measured in two directions along the cross line to obtain the average value.

The effects of different nitrogen sources in the media on the growth of the mycelia were as follows: 20 g of glucose, 1.5 g of MgSO_4_·7H_2_O, 3 g of K_2_HPO_4_, and 18 g of agar. Different nitrogen sources were then added according to the proportions of the media: 3.86 g of yeast extract, 0.59 g of urea, 2.08 g of beef extract, 1.27 g of ammonium sulfate, and 1.95 g of potassium nitrate (SCR Co., Ltd., Shanghai, China). The solution was subsequently diluted to 1 L with distilled water. A 6 mm diameter block was inoculated on different nitrogen source solid plate media and cultured in a 25 °C static incubator under dark conditions until one of the treatment groups was covered with a plate. The colony diameter and biomass were measured.

PDA medium was used to adjust the pH to 5.0, 6.0, 7.0, 8.0, and 9.0 with a 1 mol·L^−1^ NaOH solution and a 1 mol·L^−1^ HCl solution. Fungi with a diameter of 6 mm were selected and inoculated on a solid plate medium and cultured in a constant temperature incubator at 25 °C under dark conditions until one of the treatment groups was covered with a plate. Each treatment group had 3 replicates. The colony diameter and biomass were measured.

### 2.4. Analysis of the Effects of High-Temperature Stress on P. adiposa Mycelia

The strain was cultured at 25 °C for 40 days in the dark and under ventilated conditions until the mycelium reached the post-ripening stage, after which it was incubated at 25 °C, 30 °C, or 35 °C for one day and then continuously cultured for 3 days. The samples were returned to the 25 °C fruiting box for 3 days, after which the fruiting experiment was carried out. The emergence time of the primordium (calculated from the day of the fruiting experiment) and the number of primordia were recorded, and the fruiting conditions of the different treatment groups were compared. Each treatment group had 5 replicates.

The water content in the cultivation media (40% sawdust, 30% corn straw, 17% corn flour, 10% bran, 1% gypsum, 1% lime, 0.5% NaCl, 0.3% KH_2_PO_4_, and 0.2% MgSO_4_) was set to 50%, 65%, and 80%, respectively. After the reaction was completed, the mixture was sterilized at 121 °C for 90 min in a high-pressure steam sterilizer (Panasonic, Shanghai, China). After cooling, the same liquid strain was quantitatively inoculated to observe the growth status, and the feeding depth was recorded until one of the treatment groups was full. The mushrooms were placed in a mushroom box to observe their condition, and the time of the primordium (measured from the day of the mushroom experiment), the number of primordia, and the biological efficiency (BE) were recorded. Each treatment group had 5 replicates. BE is calculated via the following equation:

BE = Ffw/Sdw × 100%(1)

where Ffw is the harvest weight per bag of fresh mushroom and Sdw is the dry weight per bag.

### 2.5. P. adiposa Cultivation

The methods of Rong et al. [16] were used. The cultivation formula is shown in Table 1. After the reaction was completed, the mixture was sterilized in a high-pressure steam sterilizer at 121 °C for 90 min. After cooling, the liquid strains were quantitatively inoculated on the test cultivation materials with a pipette (Shunyou Biotechnology Co., Ltd., Shanghai, China). Each treatment group had 3 replicates. The growth rate, biological efficiency, primordium time, primordium number, fruiting body height, pileus diameter, pileus thickness, and stipe width were recorded (Table 2).

### 2.6. Determination of the Physiological and Biochemical Indices of P. adiposa Mycelia

The strains were cultured at 25 °C for 30–45 days in the dark and ventilated to reach the post-ripening stage; subsequently, they were incubated at 25 °C, 30 °C, or 35 °C for one day and then continuously cultured for 3 days. The mixture of mycelium and cultivated material was tested every day. Each treatment group had 3 replicates.

A total of 0.1 g of the sample was taken in a sterile mortar, and 2 mL of 10% trichloroacetic acid (TCA) (SCR Co., Ltd., Shanghai, China) was added for grinding. During the grinding process, 8 mL of 10% TCA was added continuously until it was homogenized. The obtained liquid was collected by centrifugation at 4 °C for 10 min at 10,000 rpm. Then, 2 mL of the supernatant was collected, 2 mL of 0.67% thiobarbituric acid (TBA) (SCR Co., Ltd., Shanghai, China) was added, and the mixture was mixed in a boiling water bath for 15 min. Another 2 mL of distilled water was added to 2 mL of 0.67% TBA for the above treatment as a blank control. The absorbance values at 450 nm (OD450), 532 nm (OD532), and 600 nm (OD600) were measured via a UV spectrophotometer (Thermo Scientific, Shanghai, China), and the malondialdehyde (MDA) content was calculated according to the difference in absorbance. The MDA concentration (cMDA, μmol·L^−1^) was calculated according to the following formula:

cMDA = 6.45 × (OD532 − OD600) − 0.56 × OD450(2)

Superoxide dismutase (SOD) activity was determined: The samples were obtained according to the methods described in Section 2.4. An SOD-NBT kit from Suzhou Grace Biotechnology Co., Ltd. (Suzhou, China), was used to determine SOD activity (product code: G0103F). Catalase activity was determined: Catalase (CAT) activity was measured via a CAT kit from Suzhou Grace Biotechnology Co., Ltd. (product code: G0105F). Glutathione reductase (GR) activity was determined: GR activity was measured via Solarbio Biotechnology Co., Ltd. (Beijing, China), and a GR activity assay kit (product code: BC1160) was used. Cellulase activity was determined: cellulase activity was measured via LEAGENE’s cellulase detection kit (DNS microplate method, product code: TE0189).

Laccase activity was determined: A 0.1 g sample was ground in a sterile mortar with 2 mL of citric acid–disodium hydrogen phosphate buffer (pH = 5.0). During the grinding process, 8 mL of buffer was added continuously until it was homogenized. The obtained liquid was collected by centrifugation and centrifuged at 4 °C for 10 min at 10,000 rpm to obtain the supernatant, namely the crude enzyme mixture. To 0.05 mL of the crude enzyme mixture, we added 1.95 mL of buffer and 1 mL of 1 mmol·L^−1^ 2,2′-azino-bis(3-ethylbenzothiazoline-6-sulfonic acid) (ABTS) (SCR Co., Ltd., Shanghai, China) to make a 3.0 mL reaction system. For the blank control group, 0.05 mL of deionized water was used instead of the crude enzyme mixture. After the reaction system reacted at 25 °C for 3 min, the absorbance was measured at a wavelength of 420 nm, and three replicates were performed for each group.

### 2.7. Method and Index Determination of Solid-State Fermentation of Cereals by P. adiposa

The method of Thomas et al. [17] was used. Fermentation method and sample treatment were as follows: On a clean bench, 5 mL of the liquid strain was inoculated into the grain medium and cultured in the dark at 25 °C. During fermentation, samples were taken every 7 days to determine the location where the hyphae spread. Fermentation lasted for 28 days. The samples were dried overnight at 60 °C, ground, and sifted through a 0.4 mm sieve.

Total phenol content was determined: A UV spectrophotometer was used to determine the total phenol content. The total phenolic content of the fermented grain samples was determined via the Folin–Ciocalteu colorimetric method. Gallic acid (SCR Co., Ltd., Shanghai, China) was used as the standard to establish a calibration curve, and the results are expressed as gallic acid equivalents (GAEs) per gram of fermented grain. Briefly, the fermented grains were first homogenized, and 2.00 g of the homogenate was extracted with 12 mL of 80% ethanol by ultrasonic treatment at 30 °C for 2 h. The mixture was filtered through qualitative filter paper, and the extraction was repeated once. The two filtrates were combined. Next, 0.1 mL of the combined extract was transferred into a 2 mL EP tube, followed by the addition of 0.2 mL of 10% Folin–Ciocalteu reagent (SCR Co., Ltd., Shanghai, China). The mixture was vortexed to ensure a complete reaction between the extract and the reagent. Subsequently, 0.8 mL of sodium carbonate solution (Na_2_CO_3_, 0.7 mol·L^−1^) was added, and the reaction mixture was incubated at 25 °C for 2 h. For the preparation of the standard curve, 0.2 mL of gallic acid solutions with concentrations of 0, 0.05, 0.1, 0.15, 0.2, and 0.25 mg·mL^−1^ were treated under the same conditions. The absorbances of both the standards and the samples were measured at 765 nm via a UV-visible spectrophotometer (ThermoScientific, Shanghai, China). The total phenolic content in the fermented grains was calculated on the basis of the calibration curve and expressed as milligrams of gallic acid equivalents per gram of fermented grain.

The reducing sugar content and DPPH free radical scavenging activity were determined via colorimetry. The reducing sugar content in the fermented grain samples was quantified via the 2,4-dinitrosalicylic acid (DNS) colorimetric method. A glucose standard curve was first established. Standard glucose solutions (1 mg·mL^−1^) of 0, 0.2, 0.4, 0.6, 0.8, 1.0, and 1.2 mL were added to a series of test tubes, followed by the addition of 2.0, 1.8, 1.6, 1.4, 1.2, 1.0, and 0.8 mL of distilled water, respectively. Subsequently, 1.5 mL of DNS reagent was added to each tube. The mixtures were thoroughly shaken and heated in a boiling water bath for 5 min and then cooled to room temperature. The absorbance was measured at 540 nm in a tube containing 0 mL of glucose as a blank. A standard curve was plotted with the absorbance values on the y-axis and the corresponding mass of glucose on the x-axis, from which a linear regression equation was derived. For sample analysis, 0.1 g of pretreated fermented grain was weighed and extracted with 2 mL of distilled water in a thermostatic water bath at 80 °C for 30 min. After centrifugation, 0.4 mL of the resulting supernatant was mixed with 0.3 mL of DNS reagent in a 2 mL centrifuge tube. The mixture was then treated under the same conditions as those used for standard curve preparation. The absorbance at 540 nm was recorded, and each treatment was replicated three times. The reducing sugar content in the fermented grain samples was calculated on the basis of the absorbance values and the standard calibration curve.

Soluble protein content was determined: A standard curve was generated in which 1 mg·mL^−1^ standard protein solution of 0, 0.1, 0.2, 0.4, 0.6, 0.8, or 1.0 mL was added to the test tube, and 1 mL was supplemented with PBS buffer (SCR, Shanghai, China). Five milliliters of Coomassie brilliant blue reagent was added to each test tube, and each sample was mixed immediately. The absorbance was measured immediately after 2 min of reaction at room temperature. The standard curve was generated with the protein content as the abscissa and the absorbance at OD595 as the ordinate. After screening, 1.0 g of each sample was removed and ground with 2 mL of PBS buffer. During the grinding process, 8 mL of PBS buffer was added continuously until it was homogenized. The obtained liquid was collected by centrifugation at a speed of 10,000 rpm·min^−1^ and centrifuged at 4 °C for 10 min. The supernatant was taken as the protein extract. Then, 0.1 mL of protein extract was mixed with 5 mL of Coomassie brilliant blue reagent (Thermo Scientific, Shanghai, China), and the absorbance was measured immediately after the reaction at room temperature for 2 min. The blank control used PBS buffer instead of protein extract. Each treatment group had 3 replicates.

### 2.8. Statistical Analysis

All the experiments were conducted in accordance with a completely randomized design, and each treatment was repeated three times. SPSS software v 19.0 was used to analyze the data.

## 3. Results

### 3.1. Pure Culture and Morphological Characteristics of P. adiposa Mycelia

The fruiting body of *P. adiposa* is composed of a pileus, stipe, and ring (Figure 1A). The colonies on the PDA plate were regular and round, with a cotton-like appearance and a loose texture (Figure 1B). The mycelium was regular in shape and could form many short, rod-shaped, colorless, transparent spores with a diameter of 2–4 μm (Figure 1B). Basidiospores were produced approximately 3 days after plate culture. Basidiospores were smooth and oval in shape and 3~5.5 μm long × 1~3.7 μm wide (Figure 1B). The sequence similarity between the isolated and purified strains and the related sequence of *P. adiposa* was as high as 98%, and the strain was identified as *P. adiposa* (Figure 1C). During maturation, yellow-brown pigments are secreted to form yellow fruiting bodies (Figure 1D). The diameter of the pileus was 1–6 cm. The initial formation of the pileus was hemispherical, and the edge was involuted. The color is yellowish brown or golden yellow, and the surface has triangular scales arranged in convex concentric rings. The stipes are 3–7 cm in length and 0.3–1.3 cm in width, and most of them are equal in thickness.

### 3.2. Effects of Different Culture Conditions on the Growth Characteristics of P. adiposa Mycelia

**Figure 2:**
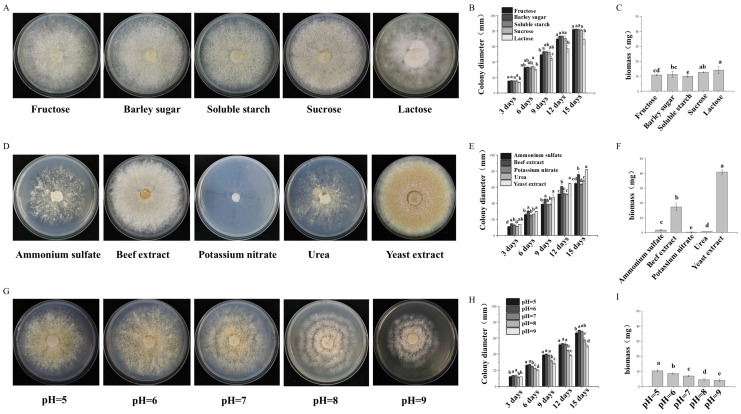
The effects of different culture conditions on the growth characteristics of *P. adiposa* mycelia. (**A**) The growth of *P. adiposa* mycelia in the presence of different carbon sources. (**B**) The colony diameter of *P. adiposa* mycelia cultured in media supplemented with different carbon sources. (**C**) Biomass statistics of *P. adiposa* mycelia grown in media supplemented with different carbon sources. (**D**) The growth of *P. adiposa* mycelia in media supplemented with different nitrogen sources. (**E**) The colony diameter of *P. adiposa* mycelia cultured in media supplemented with different nitrogen sources. (**F**) Biomass statistics of *P. adiposa* mycelia grown in media supplemented with different nitrogen sources. (**G**) The growth of *P. adiposa* mycelia at different pH values. (**H**) The colony diameter of *P. adiposa* mycelia cultured at different pH values. (**I**) Biomass statistics of *P. adiposa* mycelia at different pH values. *p* values were calculated by Student’s *t* test, a–e is *p* < 0.05.

The factors influencing *P. adiposa* mycelial growth were explored. We inoculated the plants with different carbon sources, different nitrogen sources, and different pH conditions. The results showed that *P. adiposa* could use a variety of carbon sources and grow on media supplemented with different carbon sources. The growth morphology of *P. adiposa* was the best on media supplemented with sucrose as a carbon source, and the mycelia were dense (Figure 2A). The colony diameter spread fastest on media supplemented with maltose as the carbon source, and the colony diameter on the 15th day was 82.47 ± 0.62 mm. The colony diameters in the fructose, soluble starch, sucrose, and lactose treatment groups were 82.17 ± 0.94 mm, 81.96 ± 1.88 mm, 81.42 ± 0.86 mm, and 69.54 ± 6.91 mm, respectively (Figure 2B). The biomass was the highest on the medium with lactose as the carbon source, reaching 14.08 ± 2.44 mg on the 15th day. When sucrose, maltose, fructose, and soluble starch were added as carbon sources, the biomass was 12.66 ± 0.35 mg, 11.27 ± 1.94 mg, 10.87 ± 0.42 mg, and 9.99 ± 0.80 mg, respectively (Figure 2C).

The culture results for different nitrogen sources revealed that *P. adiposa* could use a variety of nitrogen sources and grow on media supplemented with different nitrogen sources. The colony morphology of *P. adiposa* on medium supplemented with beef extract as a nitrogen source was the best, the mycelia were dense, and the color was white (Figure 2D). The colony diameter spread fastest on media supplemented with yeast powder. The colony diameter on the 15th day was 81.93 ± 0.20 mm, followed by beef paste, urea, ammonium sulfate, and potassium nitrate. The colony diameters were 75.7 ± 4.08 mm, 68.32 ± 1.09 mm, 64.83 ± 1.29 mm, and 63.68 ± 0.90 mm, respectively (Figure 2E). The biomass was greatest on media supplemented with yeast extract powder, reaching 41.04 ± 1.58 mg on the 15th day, while the biomasses of beef extract, ammonium sulfate, urea, and potassium nitrate were 17.33 ± 2.70 mg, 1.72 ± 0.51 mg, 0.73 ± 0.05 mg, and 0.31 ± 0.12 mg, respectively (Figure 2F).

The results of the cultivation of different nitrogen sources revealed that mycelia could grow from pH = 5 to pH = 9. With increasing pH, the growth potential of mycelia first increased but then decreased, and the growth potential was greatest at pH 6 (Figure 2G). The colony diameter first increased but then decreased with increasing pH. On the 15th day, the colony diameters from pH 5 to 9 were 66.54 ± 1.92 mm, 70.05 ± 0.28 mm, 68.28 ± 1.00 mm, 57.84 ± 2.81 mm, and 50.11 ± 1.90 mm, respectively. At pH = 6, the colony diameter decreased the fastest (Figure 2H). The colony biomass gradually decreased with increasing pH. On the 15th day, the biomasses from pH 5 to 9 were 10.40 ± 0.82 mg, 8.64 ± 0.46 mg, 7.05 ± 0.53 mg, 4.51 ± 0.82 mg, and 4.17 ± 0.87 mg, respectively. The biomass was greatest at pH 5 (Figure 2I).

### 3.3. The Influence Mechanism of Temperature and Moisture on P. adiposa Mycelia

**Figure 3:**
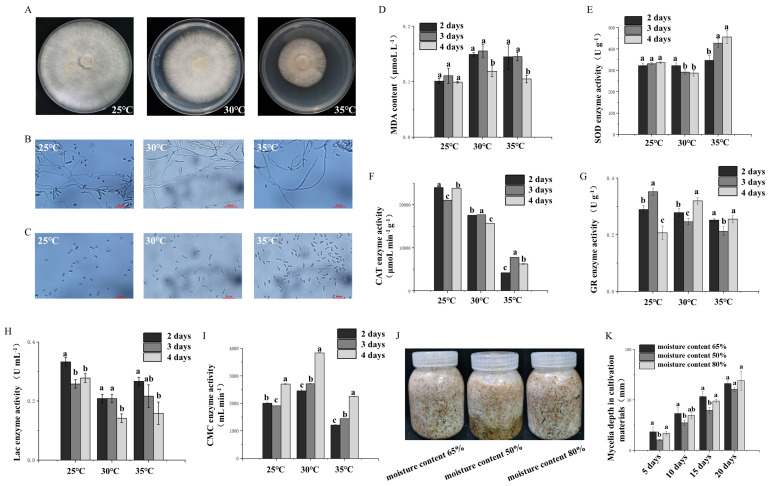
The mechanism of the effects of different temperatures and humidities on the mycelia of *P. adiposa*. (**A**–**C**) The growth status of *P. adiposa* mycelia at different temperatures. (**D**) Changes in the MDA content of *P. adiposa* mycelia at different temperatures. (**E**) Changes in SOD activity in *P. adiposa* mycelia at different temperatures. (**F**) Changes in CAT enzyme activity in *P. adiposa* mycelia at different temperatures. (**G**) Changes in the GR content in *P. adiposa* mycelia at different temperatures. (**H**) Changes in laccase activity in *P. adiposa* mycelia at different temperatures. (**I**) Changes in cellulase activity in *P. adiposa* mycelia at different temperatures. (**J**) The effects of the moisture content of different cultivation materials on the growth of *P. adiposa* mycelia. (**K**) The effects of different moisture contents on the growth depth of *P. adiposa* mycelia. *p* values were calculated by Student’s *t* test, a–c is *p* < 0.05.

To explore the effects of ambient temperature and moisture on the growth of *P. adiposa* mycelia, *P. adiposa* mycelia were cultured at three temperatures: 25 °C, 30 °C, and 35 °C (Figure 3A). The results revealed that the mycelia grew normally at 25 °C, grew slowly at 30 °C, and grew slowly at 35 °C. The two mycelia treated with high temperature did not die under high-temperature stress and could continue to grow after recovering to the appropriate temperature. To further explore the reason why high temperature affects the growth of mycelia, we observed the structure and sporulation characteristics of the mycelia via microscopy. The results showed that high-temperature stress could change the structure of hyphae. The branches of hyphae under high-temperature stress at 30 °C decreased, and the sporulation structure decreased. The branches of hyphae under high-temperature stress at 35 °C were almost absent, and the sporulation structure almost disappeared (Figure 3B). The number and volume of basidiospores increased after high-temperature stress (Figure 3C).

To further explore the mechanism underlying the effect of high temperature on *P. adiposa* mycelia. We determined the stress-related indicators and enzyme activities of the mycelia of *P. adiposa*. The concentrations of MDA in mycelia at 25 °C were 0.101 ± 0.01 μmol·L^−1^, 0.111 ± 0.01 μmol·L^−1^, and 0.099 ± 0.01 μmol·L^−1^ from the second day to the fourth day, respectively. The concentrations of MDA under high-temperature stress at 30 °C were 0.149 ± 0.01 μmol·L^−1^, 0.155 ± 0.01 μmol·L^−1^, and 0.119 ± 0.01 μmol·L^−1^ from the second day to the fourth day, respectively (Figure 3D). The concentrations of MDA under high-temperature stress at 35 °C were 0.145 ± 0.02 μmol·L^−1^, 0.145 ± 0.01 μmol·L^−1^, and 0.105 ± 0.01 μmol·L^−1^ from the second day to the fourth day, respectively. On the second day after high-temperature stress at 30 °C and 35 °C, the concentration of MDA increased by 47.5% and 43.6%, respectively, compared with that at 25 °C. On the third day, the concentration of MDA increased by 40.5% and 31.5%, respectively, compared with that at 25 °C. On the fourth day, the concentration of MDA increased by 20.2% and 6.1%, respectively, compared with that at 25 °C (Figure 3D).

The results of the determination of glutathione reductase activity revealed that the enzyme activities of the mycelia treated at 25 °C from the second day to the fourth day were 0.289 ± 0.01 U·g^−1^, 0.352 ± 0.01 U·g^−1^, and 0.207 ± 0.02 U·g^−1^. On the fourth day, the enzyme activity in the 30 °C treatment group was 54.6% greater than that in the 25 °C treatment group, and the enzyme activity in the 35 °C treatment group was 23.2% greater than that in the 25 °C treatment group (Figure 3G). The laccase activity was 0.333 ± 0.01 U·mL^−1^, 0.258 ± 0.01 U·mL^−1^, and 0.278 ± 0.02 U·mL^−1^ from the second day to the fourth day at 25 °C. On the fourth day, the enzyme activity in the 30 °C treatment group was 48.9% lower than that in the 25 °C treatment group, and the enzyme activity in the 35 °C treatment group was 43.2% lower than that in the 25 °C treatment group (Figure 3H). The cellulase enzyme activity was 2004.537 ± 18.88 μg·mL^−1^·min^−1^, 1910.185 ± 7.31 μg·mL^−1^·min^−1^, and 2704.815 ± 12.91 μg·mL^−1^·min^−1^ at 25 °C from the second day to the fourth day. On the fourth day, the enzyme activity in the 30 °C treatment group was 41.8% greater than that in the 25 °C treatment group, and the enzyme activity in the 35 °C treatment group was 16.8% lower than that in the 25 °C treatment group (Figure 3I).

The growth of *P. adiposa* mycelia in the cultivation material was affected by the water content. The results showed that mycelia could grow at water contents of 50%, 65%, and 80%. However, when the water content was 65%, the mycelial growth of the forest was greatest, and a water content that was too high or too low led to a decrease in the mycelial growth rate (Figure 3J). On the 20th day, the depth of mycelium reproduction in the cultivation material with 65% water content reached 66.40 ± 2.59 mm, the depth of mycelium reproduction in the treatment group with 50% water content was 60.58 ± 1.93 mm, and the depth of mycelium reproduction in the treatment group with 80% water content was 69.14 ± 9.55 mm (Figure 3K).

### 3.4. Effects of Different Cultivation Materials on the Formation of Fruiting Bodies in P. adiposa

**Figure 4:**
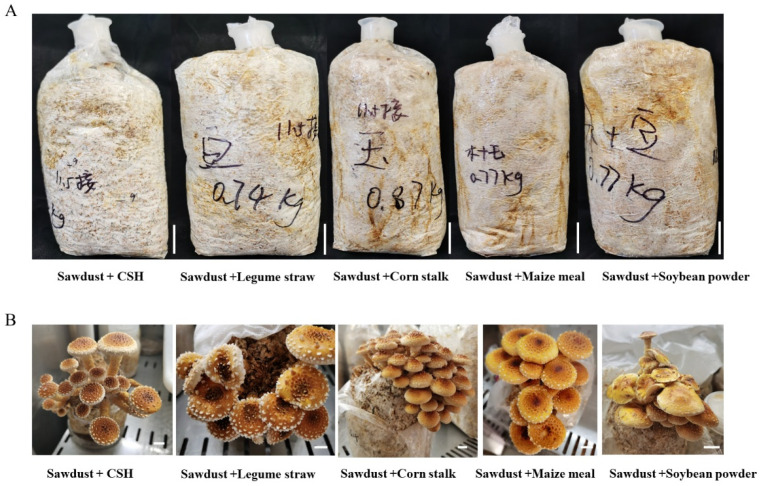
The effects of different cultivation materials on the formation of fruiting bodies in *P. adiposa*. (**A**) The growth status of *P. adiposa* mycelia in different culture media. (**B**) The morphology of fruiting bodies of *P. adiposa* in different culture materials. (Scale bar = 1 cm).

To explore the optimal culture material for the use of agricultural waste, this study carried out cultivation experiments with different culture materials. The results showed that the mycelium could grow on a variety of cultivation materials and could be widely used for the cultivation of sawdust and a variety of agricultural wastes. The mycelial growth of the *P. adiposa* strain was fastest in the sawdust + cottonseed hull cultivation group and reached 76.84 ± 1.91 mm after 20 days of culture. The remaining materials were mixed sawdust + soybean powder, mixed sawdust + corn flour, mixed sawdust + bean straw, and mixed sawdust + corn straw. After 20 days of culture, the mycelial growth depths were 76.25 ± 2.81 mm, 71.25 ± 4.74 mm, 66.48 ± 4.14 mm, and 66.40 ± 2.59 mm, respectively (Figure 4A). Mycelia were allowed to grow in bags for 40 days. A temperature range of 10–15 °C and light stimulation were applied to produce the primordium. It takes approximately 5–7 days from the present primordium to the maturation of the fruiting body.

The use of different culture materials strongly influences the cultivation characteristics of *P. adiposa*. It takes approximately 7 ± 3 days for sawdust + corn straw to present the primordium after being stimulated by temperature and light. It took 9 ± 2 days, 11 ± 3 days, 12 ± 3 days, and 14 ± 5 days for the sawdust + cottonseed hull, sawdust + soybean straw, sawdust + corn flour, and sawdust + soybean flour treatments, respectively (Table 2). The number of primordia produced by sawdust + corn straw was the greatest, reaching 103 ± 13. The numbers of primordia produced by sawdust + soybean straw, sawdust + cottonseed hull, sawdust + corn flour, and sawdust + soybean powder were 72 ± 8, 68 ± 10, 61 ± 6, and 47 ± 9, respectively. The biological efficiency of sawdust + corn straw was 72.84%, and the biological efficiencies of sawdust + soybean straw, sawdust + cottonseed hull, sawdust + corn flour, and sawdust + soybean flour were 60.42%, 46.40%, 44.48%, and 39.95%, respectively. The yield of the sawdust + corn straw cultivation material was the highest (Table 2). The average diameter of the fruiting body pileus grown on sawdust + soybean straw was 39.79 ± 5.74 mm. The diameters of the fruiting body pileus cultured on sawdust + soybean straw, sawdust + cottonseed hull, sawdust + corn flour, and sawdust + soybean flour were 24.82 ± 6.58 mm, 26.09 ± 4.55 mm, 26.53 ± 8.24 mm, and 26.93 ± 6.21 mm, respectively. There was no difference in the effects of cultivation materials on the thickness of the pileus (*p* > 0.05) (Table 2). The width of the fruiting body stipe grown on sawdust + soybean straw was 10.78 ± 1.12 mm, which was significantly greater than that of the other cultivation materials (*p* < 0.05). The length of the mixed sawdust + corn flour cultivation material was 7.61 ± 1.21 mm. There was no significant difference among the three treatment groups: mixed sawdust + soybean straw, mixed sawdust + cottonseed hull, and mixed sawdust + soybean powder. The widths of the stipe were 5.95 ± 1.16 mm, 5.39 ± 0.80 mm, and 5.83 ± 1.17 mm, respectively. The heights of the fruiting bodies of *P. adiposa* growing on sawdust + bean straw and sawdust + corn flour were 55.33 ± 7.69 mm and 56.08 ± 14.91 mm, respectively, and those of the other three groups were lower (Table 2).

### 3.5. Effects of Temperature and Moisture on Fruiting Body Formation in P. adiposa

**Figure 5:**
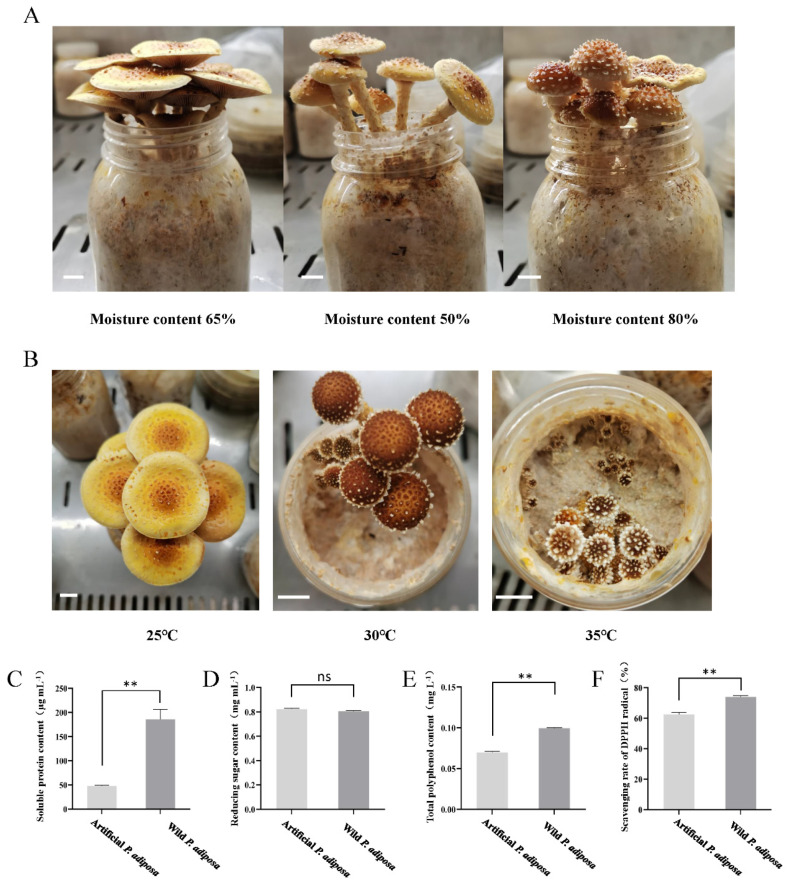
The effects of temperature and moisture on the fruiting body of *P. adiposa*. (**A**) The effects of different moisture contents on the fruiting body morphology of *P. adiposa*. (**B**) The effects of different temperatures on the fruiting body morphology of *P. adiposa*. (**C**) The determination of the soluble protein content of *P. adiposa*. (**D**) The determination of the reducing sugar content of *P. adiposa*. (**E**) The determination of the total polyphenol content of *P. adiposa*. (**F**) The determination of the scavenging rate of the DPPH radical of *P. adiposa*. (Scale bar = 1 cm). *p* values were calculated by Student’s *t* test, ** is *p* < 0.01, ns is *p* > 0.05.

To explore the effects of different temperatures and humidities on the formation of *P. adiposa* bodies, we studied the effects of three temperatures (25 °C, 30 °C, and 35 °C) and three humidities (50%, 65%, and 80%) (Figure 5A,B). The results revealed that the different water contents of the cultivation material led to a delay in the formation of the primordium of the fruiting body, and the number of primordia and the biological efficiency decreased significantly. The durations at which the primordium had 50%, 65%, and 80% water content were 10 ± 2 days, 7 ± 3 days, and 8 ± 3 days, respectively. The number of primordia was 9 ± 2, 21 ± 5 and 16 ± 4, and the biological efficiency was 53.14%, 79.63%, and 73.69%, respectively (Figure 5A,B). An excessive moisture content of cultivation materials will lead to inconsistent fruiting times of the same primordium, resulting in difficulty in harvesting. The primordium formation time of the 30 °C treatment group was longer than that of the 25 °C treatment group, and the number of primordia decreased (Table 3). There were more primordia in the 35 °C treatment group than in the 25 °C treatment group, but most of the primordia shrank and died after a period of growth (Table 3).

To explore the quality of artificially cultivated *P. adiposa*, we analyzed the differences in nutritional components between wild and artificially cultivated *P. adiposa*. The soluble protein content under artificial cultivation was significantly lower than that under natural cultivation, with soluble protein contents of 48.28 ± 1.62 μg·mL^−1^ and 186.16 ± 27.58 μg·mL^−1^, respectively (Figure 5C). There was no significant difference in the content of reducing sugars (Figure 5D). The total polyphenol content under artificial cultivation was significantly lower than that under wild cultivation (Figure 5E). The DPPH free radical scavenging ability of artificial cultivation was significantly lower than that of the wild type. The DPPH free radical scavenging ability of the artificially cultivated *P. adiposa* was 62.51% ± 0.02%, and the DPPH free radical scavenging ability of the wild type was 73.93% ± 0.01% (Figure 5F).

### 3.6. Solid-State Fermentation of P. adiposa Mycelia Increased the Nutrient Content of Common Grains and Improved Their Antioxidant Capacity

**Figure 6:**
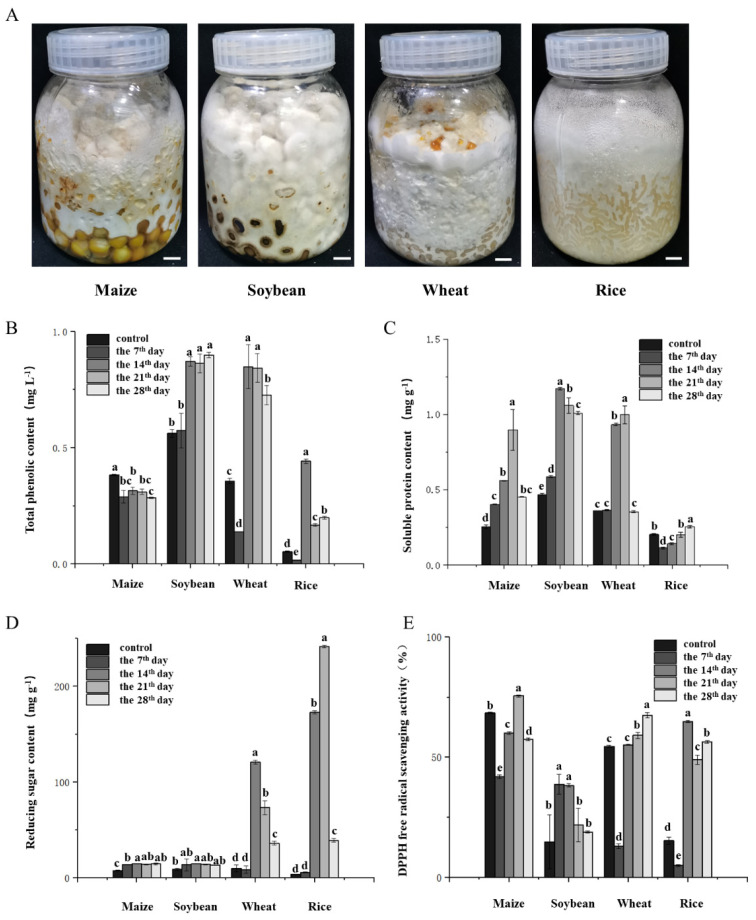
Role of *P. adiposa* mycelia in grain fermentation. (**A**) Solid-state fermentation of four cereals by *P. adiposa* mycelia. (**B**) Changes in total phenol content in four cereals by solid-state fermentation of *P. adiposa* mycelia. (**C**) Changes in soluble protein content in four cereals by solid-state fermentation of *P. adiposa* mycelia. (**D**) Changes in reducing sugar content in four cereals by solid-state fermentation of *P. adiposa* mycelia. (**E**) Changes in DPPH free radical scavenging activity in four cereals by solid-state fermentation of *P. adiposa* mycelia. *p* values were calculated by Student’s *t* test, a–e is *p* < 0.05.

To further explore the role of *P. adiposa* in the fermentation of solid grains, in this study, the mycelia of *P. adiposa* were fermented from four grains—corn, soybean, wheat, and rice—to produce many white mycelia (Figure 6A). The total phenolic content of corn was 0.384 ± 0.01 mg·L^−1^, 0.289 ± 0.03 mg·L^−1^, 0.315 ± 0.02 mg·L^−1^, 0.310 ± 0.01 mg·L^−1^, and 0.285 ± 0.01 mg·L^−1^ from day 0, day 7, day 14, day 21, and day 28, respectively, and decreased with increasing fermentation time (Figure 6B). The total phenolic content of the fermented soybean plants reached a maximum on the 28th day (0.899 ± 0.01 mg·L^−1^), which was 0.6 times greater than that of the unfermented soybean plants (0.562 ± 0.02 mg·L^−1^). Regarding the fermentation of wheat and rice by *P. adiposa*, the total phenolic content of wheat and rice reached a maximum at 14 days of fermentation at 0.849 ± 0.09 mg·L^−1^ and 0.442 ± 0.01 mg·L^−1^, respectively, which were 1.4 times and 7.4 times greater than those of the control (Figure 6B).

The soluble protein contents of the corn extract were 0.255 ± 0.01 mg·g^−1^, 0.404 ± 0.01 mg·g^−1^, 0.561 ± 0.01 mg·g^−1^, 0.898 ± 0.13 mg·g^−1^, and 0.452 ± 0.01 mg·g^−1^ from days 0, 7, 14, 21, and 28, respectively. The soluble protein content reached its highest level on day 21, which was 2.5 times greater than that without fermentation (Figure 6C). The soluble protein content of the fermented soybean plants reached a maximum on the 14th day, which was 1.5 times greater than that of the unfermented soybean plants. The soluble protein content in the wheat substrate reached its highest value on the 21st day, which was 1.8 times greater than that of the unfermented substrate. The soluble protein content in the rice matrix reached its highest value on the 28th day, which was 0.3 times greater than that of unfermented rice (Figure 6C).

The content of reducing sugars in the corn matrix reached its highest value (14.881 ± 0.12 mg·g^−1^) on the 14th day, which was double that of the unfermented corn matrix (7.615 ± 0.52 mg·g^−1^). The reducing sugar content of the fermented soybean plants reached its highest value on the 14th day (14.870 ± 0.37 mg·g^−1^), which was 0.6 times greater than that of the unfermented soybean plants (9.110 ± 1.21 mg·g^−1^). The reducing sugar content of the wheat plants reached a maximum on the 14th day (120.896 ± 1.76 mg·g^−1^), which was 11.2 times greater than that of the unfermented plants (9.944 ± 3.74 mg·g^−1^). The reducing sugar content of the fermented rice reached a maximum on the 21st day (241.632 ± 1.38 mg·g^−1^), which was 66.4 times greater than that of the unfermented rice (3.583 ± 0.07 mg·g^−1^).

To further explore whether the lipid umbrella can improve the antioxidant capacity of cereals, we measured the DPPH free radical scavenging ability of corn, soybean, wheat, and rice. The DPPH free radical scavenging ability of corn was 68.47 ± 0.34%, 41.91 ± 0.74%, 60.16 ± 0.59%, 75.63 ± 0.41%, and 57.54 ± 0.59% on the 0th, 7th, 14th, 21st, and 28th days of fermentation, respectively. The DPPH free radical scavenging ability of the fermented soybeans reached a maximum on the 7th day (38.80 ± 4.17%), which was 0.6 times greater than that of the unfermented soybeans (14.86 ± 11.22%). The DPPH free radical scavenging ability of the fermented wheat reached a maximum on the 28th day (67.49 ± 1.09%), which was 11.2 times greater than that of the unfermented wheat (54.59 ± 0.43%). The DPPH free radical scavenging ability of the fermented rice reached a maximum on the 14th day (64.92 ± 0.43%), which was 66.4 times greater than that of the unfermented rice (15.30 ± 1.52%).

## 4. Discussion

As favorite foods, macrofungi have been cultivated and eaten in China since ancient times. There are approximately 277 species of edible macrofungi in China, which are concentrated mainly in Northeast China and Southwest China [18]. The strain is the most important step in the artificial cultivation of macrofungi. As heterotrophic microorganisms, macrofungi need to be provided with nutrients from the outside world [19]. The carbon and nitrogen required for growth need external supplies. By exploring the growth characteristics of macrofungal mycelia, the best carbon source, nitrogen source, carbon-nitrogen ratio, temperature, pH value, and medium suitable for mycelial growth can be determined [20].

As a famous edible and medicinal fungus, *P. adiposa* is rich in nutrients and medicinal ingredients and has been reported to have antibacterial activity [7]. Its polysaccharide components have antioxidant activity and antitumor effects in vitro [21,22]. Lectins isolated from *P. adiposa* have been shown to have anti-proliferative effects on HepG2 and MCF7 cells [23]. Methyl gallate isolated from *P. adiposa* has antioxidant, anti-HIV-1, and anti-HIV-1 enzyme inhibitory effects [24]. However, the large-scale cultivation of *P. adiposa* has not been reported. This poses a challenge for further research on its active ingredients and drug development. In our study, we first collected fruiting bodies of wild *P. adiposa* from the Maoer Mountain Forest Farm for morphological and molecular biological identification (Figure 1C) and then cultivated *P. adiposa* in sawdust media (Figure 4B). This process laid the foundation for a large amount of cultivation and drug research and development.

The preparation of strains is the most important step in the production of macrofungi, and the quality of strains can affect the yield of macrofungi. *P. adiposa* is a kind of wood rot fungus at medium and low temperatures, and its purity and growth affect the growth of macrofungi [2,25]. High-quality strains have a high degree of purification, strong viability, strong germination ability, fast feeding speed, and high biological efficiency. However, bacterial contamination, disease infection, high-temperature stress, and excessive passage of strains affect the quality of strains. The characteristics of high-quality strains are as follows: white mycelium, uniform growth, strong resistance, and more cultivation primordia [18].

Temperature is one of the key factors affecting the growth of edible fungi. In the production process of large fungi, to save space, the bags need to be stacked together, which easily produces a large amount of heat so that the local bags are in a high-temperature environment [26,27]. However, the growth characteristics of high-quality strains do not change after exposure to high-temperature stress. Researchers usually observe and study the mycelia of macrofungi from both macro- and microscale perspectives to identify high-quality macrofungi that can resist high-temperature stress [26,28]. In this study, the number of basidiospores increased significantly after high-temperature treatment (Figure 3C).

In the face of high-temperature stress, the mycelia of *P. adiposa* resist oxidative damage by repairing their own plasma membrane and increasing the secretion of antioxidant enzymes [4]. Under high-temperature stress at 35 °C, the activity of SOD was significantly greater than that of mycelia at the optimum temperature, while the CAT activity and GR activity also increased on the 3rd day. In addition, the activities of antioxidant enzymes and extracellular enzymes in mycelia under 35 °C high-temperature stress were greater than those under 30 °C high-temperature stress, indicating that the strain may be regulated by a certain gene and secrete more antioxidant enzymes to protect itself (Figure 3D–I). However, long-term high-temperature stress strongly affects the growth rate of mycelia and stops their growth. Therefore, the culture temperature must receive close attention during the production process. Furthermore, the antioxidant enzyme activity was monitored for only 2–4 days at 35 °C, which is a relatively short period and may not fully reflect the heat tolerance of the strain. Future studies should involve longer monitoring and recovery periods to confirm the heat resistance of strains.

Edible fungi are heterotrophic eukaryotes that rely on extracellular enzymes to decompose lignin and cellulose for nutrition [27,29]. Among them, the laccase activity of *C. licheniformis* decreases at high temperatures, which affects its ability to decompose substrates. In contrast, cellulase activity increased at high temperatures, and an appropriate increase in temperature on the surface improved cellulase activity, resulting in a strong ability to adapt to high temperatures [3,30]. Lignin is widely present in the cell wall of terrestrial plants and is abundant but difficult to use. Lignin is difficult to use and has a complex structure. Under NaOH treatment, hemicellulose is degraded first, whereas lignin is degraded less [31,32]. Our study revealed that *P. adiposa* mycelia can degrade lignin and convert it into nutrients, which has important research value (Figure 4A).

Fermentation, a traditional food processing method, is widely used to process grains in Asia and Africa [33]. With the development of biotechnology, the use of microorganisms to produce commercially valuable fermentation methods has been successfully applied in the food, textile, and pharmaceutical industries [34,35,36]. The solid-state fermentation of cereals using macrofungal mycelia can not only result in a special flavor and improve the antioxidant capacity of cereals but also decompose nutrients in cereals into small molecules that are more easily absorbed by the human body [37]. Fermented grains undergo different changes in nutrient content and antioxidant capacity, which provides more suggestions for commercialization to meet the needs of consumers.

Phenolic compounds are among the most important secondary metabolites of macrofungi and have good antioxidant activity [38,39]. Our results showed that the solid-state fermentation of cereals by the mycelium of *P. adiposa* significantly increased the content of phenolic compounds (Figure 6B). Soluble protein and reducing sugars are not only the main nutrients of cereals but also affect the taste of food. Our results showed that the solid-state fermentation of cereals significantly increased the contents of soluble protein and reducing sugars (Figure 6B,C). An improvement in the DPPH superoxide radical scavenging ability represents an improvement in the antioxidant capacity. Our results showed that the fermented grains of *P. adiposa* mycelia significantly improved DPPH free radical scavenging ability. Corn and rice are rich in carbohydrates, and their antioxidant capacity is mainly concentrated in the outer skin. Soybean is an indispensable food in daily life and is rich in phenols and proteins. Wheat is rich in vitamin E and polyphenols. The contents of these four kinds of grain nutrients differ, so further research is needed to lay the foundation for a better utilization of common human grains and the improvement of grain nutrition.

This study provides a solid foundation for the large-scale application of *P. adiposa*. To enable successful industrial-scale deployment, two major challenges must be addressed: technical difficulties and cost management. To address these technical challenges, automated environmental control systems could be introduced to precisely regulate the temperature and humidity in fermentation facilities. Additionally, breeding or selecting more thermotolerant and fast-growing strains can shorten fermentation cycles, increase yields, and reduce contamination risks, thereby improving overall production efficiency. For cost control, future work should explore the use of locally available, inexpensive agricultural byproducts (such as rice husks, corn stalks, and soybean meal) as fermentation substrates and employ modular fermentation units to minimize construction and maintenance costs. To further support the scalability of this approach, a pilot-scale fermentation platform should be established that is equipped with precise temperature and humidity control systems for continuous monitoring of mycelial growth, product dynamics, and nutrient composition. Moreover, additional resources are needed to conduct long-term studies on strain thermotolerance and product consistency, optimize solid-state fermentation parameters, and perform multiple batch validations to ensure process stability and product quality control.

## 5. Conclusions

In this study, *P. adiposa* material was collected in the field, and the domestication and cultivation of high-quality mycelium were performed. The effects of high temperature on the mycelia of *P. adiposa* and the ability of *P. adiposa* mycelia to improve the antioxidant activity and the content of easily absorbed nutrients during grain fermentation were investigated. The mycelia isolated in this study can adapt to high-temperature conditions, even in a 35 °C high-temperature environment. It recovers quickly at 25 °C, which greatly improves the survival ability of the umbrella under high-temperature stress and the ability to continue to grow after recovering to the optimum temperature. Further research revealed that the mycelia of *P. adiposa* could significantly increase the total phenol content of soybean, wheat, and rice. The soluble protein content, reducing sugar content, and antioxidant capacity of corn, soybean, wheat, and rice significantly increased. Therefore, exploring the cultivation and fermentation of common grains may greatly improve social needs and increase economic benefits.

## Figures and Tables

**Table 1:** Composition of cultivation material for *P. adiposa*.

Types	Formula
Sawdust + CSH	Sawdust 40%, CSH 30%, Maize meal 17%, Wheat bran 10%, Gypsum 1%, Lime 1%, NaCl 0.5%, KH_2_PO_4_ 0.3%, MgSO_4_ 0.2%
Sawdust + Legume straw	Sawdust 40%, Legume straw 30%, Maize meal 17%, Wheat bran 10%, Gypsum 1%, Lime 1%, NaCl 0.5%, KH_2_PO_4_ 0.3%, MgSO_4_ 0.2%
Sawdust + Corn stalk	Sawdust 40%, Corn stalk 30%, Maize meal 17%, Wheat bran 10%, Gypsum 1%, Lime 1%, NaCl 0.5%, KH_2_PO_4_ 0.3%, MgSO_4_ 0.2%
Sawdust + Maize meal	Sawdust 70%, Maize meal 17%, Wheat bran 10%, Gypsum 1%, Lime 1%, NaCl 0.5%, KH_2_PO_4_ 0.3%, MgSO_4_ 0.2%
Sawdust + Soybean powder	Sawdust 70%, Soybean powder 17%, Wheat bran 10%, Gypsum 1%, Lime 1%, NaCl 0.5%, KH_2_PO_4_ 0.3%, MgSO_4_ 0.2%

Notes: CSH refers to cotton seed hull.

**Table 2:** The effects of different cultivation materials on the growth of *P. adiposa* fruiting bodies.

Culture Material	Appearance of Primordia (Day)	Number of Primordia	Biological Efficiency (%/m/m)	Cap Diameter (mm)	Cap Thickness (mm)	Stalk Width (mm)	Height of Fruiting Body (mm)
Sawdust + CSH	9.00 ± 2.00 ^a^	68.00 ± 10.00 ^c^	46.40 ^c^	24.82 ± 6.58 ^b^	6.72 ± 1.33 ^a^	5.95 ± 1.16 ^c^	45.61 ± 12.82 ^b^
Sawdust + Legume straw	11.00± 3.00 ^a^	72.00 ± 8.00 ^b^	60.42 ^b^	39.79 ± 5.74 ^a^	7.10 ± 1.10 ^a^	10.78 ± 1.12 ^a^	55.33 ± 7.69 ^a^
Sawdust + Corn stalk	7.00 ± 3.00 ^a^	103.00 ± 13.00 ^a^	72.84 ^a^	26.09 ± 4.55 ^b^	7.37 ± 0.97 ^a^	5.39 ± 0.80 ^c^	49.33 ± 7.53 ^ab^
Sawdust + Maize meal	12.00 ± 3.00 ^a^	61.00 ± 6.00 ^cd^	44.48 ^d^	26.53 ± 8.24 ^b^	6.93 ± 2.23 ^a^	7.61 ± 1.21 ^b^	56.08 ± 14.91 ^a^
Sawdust + Soybean powder	14.00 ± 5.00 ^a^	47.00 ± 9.00 ^d^	39.95 ^e^	26.93 ± 6.21 ^b^	6.61 ± 1.17 ^a^	5.83 ± 1.17 ^c^	44.52 ± 9.57 ^b^

Notes: Different letters in columns in superscripts indicate significant differences in the growth of *P. adiposa* fruiting bodies on different cultivation materials (*p* < 0.05).

**Table 3:** Effects of different temperatures and humidities on *P. adiposa* growth.

Different Treatment Groups		Appearance of Primordia (Day)	Number of Primordia
Moisture	50%	10 ± 2 ^a^	9 ± 2 ^b^
	65%	7 ± 3 ^a^	21 ± 5 ^a^
	80%	8 ± 3 ^a^	16 ± 4 ^ab^
Temperature	25 °C	7 ± 3 ^a^	21 ± 5 ^b^
	30 °C	10 ± 5 ^a^	12 ± 3 ^b^
	35 °C	15 ± 6 ^a^	33 ± 8 ^a^

Notes: Different letters in columns with superscripts indicate significant differences in *P. adiposa*. Growth under different temperature and humidity treatments.

## Data Availability

The original contributions presented in this study are included in the article. Further inquiries can be directed to the corresponding authors.
